# Supplementary figures and images for: Mapping potential risks for the transmission of spotted fever rickettsiosis: The case study from the Rio de Janeiro state, Brazil
Source: PLoS One. 2022 Jul 6;17(7):e0270837. doi: 10.1371/journal.pone.0270837 (PMC9258828; doi:10.1371/journal.pone.0270837)

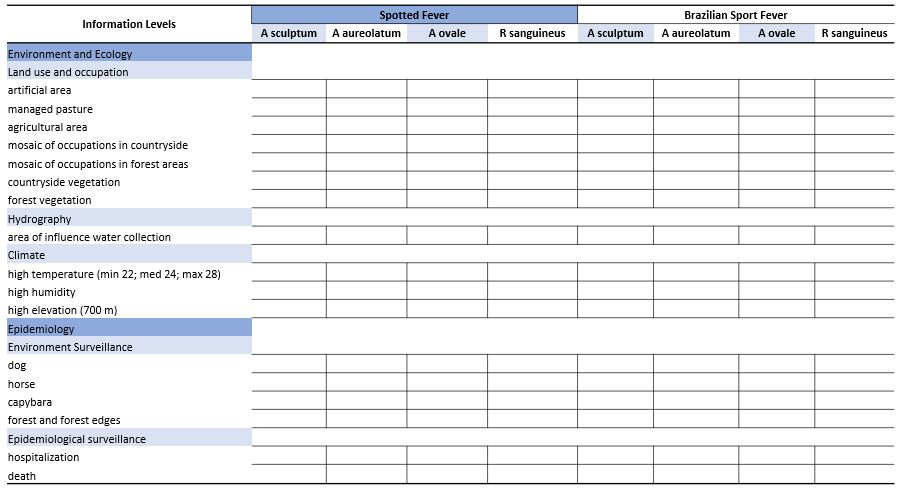

Supplement: S1 Fig — (TIF) [file pone.0270837.s001.tif]
