# Supplementary material for: Mapping potential risks for the transmission of spotted fever rickettsiosis: The case study from the Rio de Janeiro state, Brazil
Source: PLoS One. 2022 Jul 6;17(7):e0270837. doi: 10.1371/journal.pone.0270837 (PMC9258828; doi:10.1371/journal.pone.0270837)
Supplement: S1 Table — AT—Aggregate Total = all weights the same, Scenery 1—only vector Amblyomma sculptum + dog + capybara + horse, Scenery 2—only vector Amblyomma aureolatum + dog, Scenery 3—only vector Rhipicephalus sanguineus + dog, Scenery 4—only vector Amblyomma ovale + dog + capybara + horse, Scenery 5—only vector Rhipicephalus sanguineus + dog. (PDF) [file pone.0270837.s002.pdf]

Scenery	Rickettsiosis	dog	capybara	horse	Asculp	Aaureo	Aovale	Rsang
AT		1	1	1	1	1	1	1
1	BSF	1	3	4	7	0	0	0
2	BSF	1	0	0	0	1	0	0
3	BSF	2	0	0	0	0	0	2
4	SF	7	1	1	0	0	9	0
5	SF	1	0	0	0	0	0	1

Legend:

AT Aggregate Total = all weights the same
1 only vector Amblyomma sculptum + dog + capybara + horse
2 only vector Amblyomma aureolatum + dog
3 only vector Rhipicephalus sanguineus +dog
4 only vector Amblyomma ovale + dog + capybara + horse
5 only vector Rhipicephalus sanguineus +dog
