# Supplementary material for: Mapping potential risks for the transmission of spotted fever rickettsiosis: The case study from the Rio de Janeiro state, Brazil
Source: PLoS One. 2022 Jul 6;17(7):e0270837. doi: 10.1371/journal.pone.0270837 (PMC9258828; doi:10.1371/journal.pone.0270837)
Supplement: S2 Table — Legend: score vh = refers to individualized ecological relationship between vector and host, score vh total = refers to the specific relationship between vector and transmitted disease and scale of 100. (PDF) [file pone.0270837.s003.pdf]

Scenery		Vector	Host	Score vh	Score vh total
**Brazilian Spotted Fever rickettsiosis**	BSF1	A sculptum	Horse	**37**	**70**
	BSF2	A sculptum	Capybara	**26**	
	BSF3	A sculptum	Dog	**7**	
	BSF4	A aureolatum	Horse	**1**	**10**
	BSF5	A aureolatum	Capybara	**1**	
	BSF6	A aureolatum	Dog	**8**	
	BSF7	R sanguineus	Horse	**2**	**20**
	BSF8	R sanguineus	Capybara	**2**	
	BSF9	R sanguineus	Dog	**16**	
**Spotted Fever rickettsiosis**	SF1	A ovale	Horse	**9**	**90**
	SF2	A ovale	Capybara	**9**	
	SF3	A ovale	Dog	**72**	
	SF4	R sanguineus	Horse	**1**	**10**
	SF5	R sanguineus	Capybara	**1**	
	SF6	R sanguineus	Dog	**8**	

Scale of 100 (%)

Legend:

score vh = refers to individualized ecological relationship between vector and host

score vh total = refers to the specific relationship between vector and transmitted disease
