# Supplementary material for: Mapping potential risks for the transmission of spotted fever rickettsiosis: The case study from the Rio de Janeiro state, Brazil
Source: PLoS One. 2022 Jul 6;17(7):e0270837. doi: 10.1371/journal.pone.0270837 (PMC9258828; doi:10.1371/journal.pone.0270837)
Supplement: S3 Table — Asculp—Amblyomma sculptum, Aaureo—Amblyomma aureolatum, Aovale—Amblyomma ovale and Rsang—Rhipicephalus sanguineus. (PDF) [file pone.0270837.s004.pdf]

| Geoenvironmental Domains | | | | | | | | | | | | | | | | | | | | | | | | | | | | | | | | | | | |
|---|---|---|---|---|---|---|---|---|---|---|---|---|---|---|---|---|---|---|---|---|---|---|---|---|---|---|---|---|---|---|---|---|---|---|---|
| Domain I - Coastal Strip | | | | | | | | | | | | Domain II - Mountain Region | | | | | | | | Domain III - Plateau of the Mountain Region | | | | Domain IV - Middle Depression Paraíba do Sul | | | | Domain V - North and Northwest Depression | | | | Domain VI - Alto Itabapoana Plateau | | | |
| Metropolitan Region | | | | Lakes Region and East Coast | | | | North Fluminense | | | | South Coast | | | | Mar and Mantiqueira Mountain Range | | | | | | | | | | | | | | | | | | | |
| A sculp | A aureo | A ovale | R sang | A sculp | A aureo | A ovale | R sang | A sculp | A aureo | A ovale | R sang | A sculp | A aureo | A ovale | R sang | A sculp | A aureo | A ovale | R sang | A sculp | A aureo | A ovale | R sang | A sculp | A aureo | A ovale | R sang | A sculp | A aureo | A ovale | R sang | A sculp | A aureo | A ovale | R sang |
| 6 | 6 | 9 | 25 | 6 | 6 | 9 | 25 | 6 | 6 | 9 | 25 | 8 | 6 | 15 | 25 | 6 | 15 | 6 | 25 | 4 | 12 | 6 | 25 | 6 | 6 | 6 | 25 | 6 | 6 | 6 | 25 | 6 | 6 | 6 | 25 |
| 9 | 1 | 1 | 1 | 12 | 1 | 1 | 1 | 12 | 1 | 1 | 1 | 12 | 1 | 1 | 1 | 12 | 1 | 1 | 1 | 9 | 1 | 1 | 1 | 12 | 1 | 1 | 1 | 12 | 1 | 1 | 1 | 12 | 1 | 1 | 1 |
| 20 | 1 | 1 | 1 | 25 | 1 | 1 | 1 | 25 | 1 | 1 | 1 | 25 | 1 | 1 | 1 | 25 | 1 | 1 | 1 | 25 | 1 | 1 | 1 | 25 | 1 | 1 | 1 | 25 | 1 | 1 | 1 | 25 | 1 | 1 | 1 |
| 9 | 4 | 6 | 20 | 9 | 4 | 6 | 20 | 12 | 4 | 6 | 20 | 12 | 4 | 10 | 20 | 9 | 10 | 4 | 20 | 6 | 8 | 4 | 20 | 9 | 4 | 4 | 20 | 9 | 4 | 4 | 20 | 9 | 4 | 4 | 20 |
| 12 | 1 | 1 | 1 | 15 | 1 | 1 | 1 | 16 | 1 | 1 | 1 | 16 | 1 | 1 | 1 | 16 | 1 | 1 | 1 | 12 | 1 | 1 | 1 | 16 | 1 | 1 | 1 | 16 | 1 | 1 | 1 | 16 | 1 | 1 | 1 |
| 20 | 1 | 1 | 1 | 25 | 1 | 1 | 1 | 25 | 1 | 1 | 1 | 25 | 1 | 1 | 1 | 25 | 1 | 1 | 1 | 25 | 1 | 1 | 1 | 25 | 1 | 1 | 1 | 25 | 1 | 1 | 1 | 25 | 1 | 1 | 1 |
| 3 | 4 | 6 | 20 | 9 | 4 | 6 | 20 | 3 | 4 | 6 | 20 | 4 | 4 | 10 | 20 | 3 | 10 | 4 | 20 | 2 | 8 | 4 | 20 | 3 | 4 | 4 | 20 | 3 | 4 | 4 | 20 | 3 | 4 | 4 | 20 |
| 9 | 1 | 1 | 1 | 12 | 1 | 1 | 1 | 12 | 1 | 1 | 1 | 12 | 1 | 1 | 1 | 12 | 1 | 1 | 1 | 9 | 1 | 1 | 1 | 12 | 1 | 1 | 1 | 12 | 1 | 1 | 1 | 12 | 1 | 1 | 1 |
| 16 | 1 | 1 | 1 | 20 | 1 | 1 | 1 | 20 | 1 | 1 | 1 | 20 | 1 | 1 | 1 | 20 | 1 | 1 | 1 | 20 | 1 | 1 | 1 | 20 | 1 | 1 | 1 | 20 | 1 | 1 | 1 | 20 | 1 | 1 | 1 |
| 9 | 8 | 12 | 25 | 9 | 8 | 12 | 25 | 9 | 8 | 12 | 25 | 12 | 8 | 20 | 25 | 9 | 20 | 8 | 25 | 6 | 16 | 8 | 25 | 9 | 8 | 8 | 25 | 9 | 8 | 8 | 25 | 9 | 8 | 8 | 25 |
| 12 | 1 | 1 | 1 | 16 | 1 | 1 | 1 | 16 | 1 | 1 | 1 | 16 | 1 | 1 | 1 | 16 | 1 | 1 | 1 | 12 | 1 | 1 | 1 | 16 | 1 | 1 | 1 | 16 | 1 | 1 | 1 | 16 | 1 | 1 | 1 |
| 20 | 1 | 1 | 1 | 25 | 1 | 1 | 1 | 25 | 1 | 1 | 1 | 25 | 1 | 1 | 1 | 25 | 1 | 1 | 1 | 25 | 1 | 1 | 1 | 25 | 1 | 1 | 1 | 25 | 1 | 1 | 1 | 25 | 1 | 1 | 1 |
| 6 | 10 | 15 | 25 | 18 | 10 | 15 | 25 | 6 | 10 | 15 | 25 | 8 | 10 | 25 | 25 | 6 | 20 | 10 | 25 | 4 | 20 | 10 | 25 | 6 | 10 | 10 | 25 | 6 | 10 | 10 | 25 | 6 | 10 | 10 | 25 |
| 9 | 1 | 1 | 1 | 12 | 1 | 1 | 1 | 12 | 1 | 1 | 1 | 12 | 1 | 1 | 1 | 12 | 1 | 1 | 1 | 9 | 1 | 1 | 1 | 12 | 1 | 1 | 1 | 12 | 1 | 1 | 1 | 12 | 1 | 1 | 1 |
| 16 | 1 | 1 | 1 | 20 | 1 | 1 | 1 | 20 | 1 | 1 | 1 | 20 | 1 | 1 | 1 | 20 | 1 | 1 | 1 | 20 | 1 | 1 | 1 | 20 | 1 | 1 | 1 | 20 | 1 | 1 | 1 | 20 | 1 | 1 | 1 |
| 6 | 8 | 12 | 20 | 18 | 8 | 12 | 20 | 6 | 8 | 12 | 20 | 8 | 8 | 20 | 20 | 6 | 20 | 8 | 20 | 4 | 16 | 8 | 20 | 6 | 8 | 8 | 20 | 6 | 8 | 8 | 20 | 6 | 8 | 8 | 20 |
| 6 | 1 | 1 | 1 | 8 | 1 | 1 | 1 | 8 | 1 | 1 | 1 | 8 | 1 | 1 | 1 | 8 | 1 | 1 | 1 | 6 | 1 | 1 | 1 | 8 | 1 | 1 | 1 | 8 | 1 | 1 | 1 | 8 | 1 | 1 | 1 |
| 16 | 1 | 1 | 1 | 20 | 1 | 1 | 1 | 20 | 1 | 1 | 1 | 20 | 1 | 1 | 1 | 20 | 1 | 1 | 1 | 20 | 1 | 1 | 1 | 20 | 1 | 1 | 1 | 20 | 1 | 1 | 1 | 20 | 1 | 1 | 1 |
| 6 | 10 | 15 | 15 | 18 | 10 | 15 | 15 | 6 | 10 | 15 | 15 | 8 | 10 | 25 | 15 | 6 | 25 | 10 | 15 | 4 | 20 | 10 | 15 | 6 | 10 | 10 | 15 | 6 | 10 | 10 | 15 | 6 | 10 | 10 | 15 |
| 6 | 1 | 1 | 1 | 8 | 1 | 1 | 1 | 8 | 1 | 1 | 1 | 8 | 1 | 1 | 1 | 8 | 1 | 1 | 1 | 6 | 1 | 1 | 1 | 8 | 1 | 1 | 1 | 8 | 1 | 1 | 1 | 8 | 1 | 1 | 1 |
| 8 | 1 | 1 | 1 | 10 | 1 | 1 | 1 | 10 | 1 | 1 | 1 | 10 | 1 | 1 | 1 | 10 | 1 | 1 | 1 | 10 | 1 | 1 | 1 | 10 | 1 | 1 | 1 | 10 | 1 | 1 | 1 | 10 | 1 | 1 | 1 |
