# Supplementary material for: Mapping potential risks for the transmission of spotted fever rickettsiosis: The case study from the Rio de Janeiro state, Brazil
Source: PLoS One. 2022 Jul 6;17(7):e0270837. doi: 10.1371/journal.pone.0270837 (PMC9258828; doi:10.1371/journal.pone.0270837)
Supplement: S4 Table — (PDF) [file pone.0270837.s005.pdf]

| Coverage and land use | | | | | | | | | | | | | | | | | | | | |
|---|---|---|---|---|---|---|---|---|---|---|---|---|---|---|---|---|---|---|---|---|
| artificial area | | | managed pasture | | | agricultural area | | | field mosaics | | | forest mosaics | | | field vegetation | | | forest vegetation | | |
| dog | capybara | horse | dog | capybara | horse | dog | capybara | horse | dog | capybara | horse | dog | capybara | horse | dog | capybara | horse | dog | capybara | horse |
| 6 | 9 | 20 | 9 | 12 | 20 | 3 | 9 | 16 | 9 | 12 | 20 | 6 | 9 | 16 | 6 | 6 | 16 | 6 | 6 | 8 |
| 6 | 1 | 1 | 4 | 1 | 1 | 4 | 1 | 1 | 8 | 1 | 1 | 10 | 1 | 1 | 8 | 1 | 1 | 10 | 1 | 1 |
| 9 | 1 | 1 | 6 | 1 | 1 | 6 | 1 | 1 | 12 | 1 | 1 | 15 | 1 | 1 | 12 | 1 | 1 | 15 | 1 | 1 |
| 25 | 1 | 1 | 20 | 1 | 1 | 20 | 1 | 1 | 25 | 1 | 1 | 25 | 1 | 1 | 20 | 1 | 1 | 15 | 1 | 1 |
| 6 | 12 | 25 | 9 | 15 | 25 | 9 | 12 | 20 | 9 | 16 | 25 | 18 | 12 | 20 | 18 | 8 | 20 | 18 | 8 | 10 |
| 6 | 1 | 1 | 4 | 1 | 1 | 4 | 1 | 1 | 8 | 1 | 1 | 10 | 1 | 1 | 8 | 1 | 1 | 10 | 1 | 1 |
| 9 | 1 | 1 | 6 | 1 | 1 | 6 | 1 | 1 | 12 | 1 | 1 | 15 | 1 | 1 | 12 | 1 | 1 | 15 | 1 | 1 |
| 25 | 1 | 1 | 20 | 1 | 1 | 20 | 1 | 1 | 25 | 1 | 1 | 25 | 1 | 1 | 20 | 1 | 1 | 15 | 1 | 1 |
| 6 | 12 | 25 | 12 | 16 | 25 | 3 | 12 | 20 | 9 | 16 | 25 | 6 | 12 | 20 | 6 | 8 | 20 | 6 | 8 | 10 |
| 6 | 1 | 1 | 4 | 1 | 1 | 4 | 1 | 1 | 8 | 1 | 1 | 10 | 1 | 1 | 8 | 1 | 1 | 10 | 1 | 1 |
| 9 | 1 | 1 | 6 | 1 | 1 | 6 | 1 | 1 | 12 | 1 | 1 | 15 | 1 | 1 | 12 | 1 | 1 | 15 | 1 | 1 |
| 25 | 1 | 1 | 20 | 1 | 1 | 20 | 1 | 1 | 25 | 1 | 1 | 25 | 1 | 1 | 20 | 1 | 1 | 15 | 1 | 1 |
| 8 | 12 | 25 | 12 | 16 | 25 | 4 | 12 | 20 | 12 | 16 | 25 | 8 | 12 | 20 | 8 | 8 | 20 | 8 | 8 | 10 |
| 6 | 1 | 1 | 4 | 1 | 1 | 4 | 1 | 1 | 8 | 1 | 1 | 10 | 1 | 1 | 8 | 1 | 1 | 10 | 1 | 1 |
| 15 | 1 | 1 | 10 | 1 | 1 | 10 | 1 | 1 | 20 | 1 | 1 | 25 | 1 | 1 | 20 | 1 | 1 | 25 | 1 | 1 |
| 25 | 1 | 1 | 20 | 1 | 1 | 20 | 1 | 1 | 25 | 1 | 1 | 25 | 1 | 1 | 20 | 1 | 1 | 15 | 1 | 1 |
| 6 | 12 | 25 | 9 | 16 | 25 | 3 | 12 | 20 | 9 | 16 | 25 | 6 | 12 | 20 | 6 | 8 | 20 | 6 | 8 | 10 |
| 15 | 1 | 1 | 10 | 1 | 1 | 10 | 1 | 1 | 20 | 1 | 1 | 20 | 1 | 1 | 20 | 1 | 1 | 25 | 1 | 1 |
| 6 | 1 | 1 | 4 | 1 | 1 | 4 | 1 | 1 | 8 | 1 | 1 | 10 | 1 | 1 | 8 | 1 | 1 | 10 | 1 | 1 |
| 25 | 1 | 1 | 20 | 1 | 1 | 20 | 1 | 1 | 25 | 1 | 1 | 25 | 1 | 1 | 20 | 1 | 1 | 15 | 1 | 1 |
| 4 | 9 | 25 | 6 | 12 | 25 | 2 | 9 | 20 | 6 | 12 | 25 | 4 | 9 | 20 | 4 | 6 | 20 | 4 | 6 | 10 |
| 12 | 1 | 1 | 8 | 1 | 1 | 8 | 1 | 1 | 16 | 1 | 1 | 20 | 1 | 1 | 16 | 1 | 1 | 20 | 1 | 1 |
| 6 | 1 | 1 | 4 | 1 | 1 | 4 | 1 | 1 | 8 | 1 | 1 | 10 | 1 | 1 | 8 | 1 | 1 | 10 | 1 | 1 |
| 25 | 1 | 1 | 20 | 1 | 1 | 20 | 1 | 1 | 25 | 1 | 1 | 25 | 1 | 1 | 20 | 1 | 1 | 15 | 1 | 1 |
| 6 | 12 | 25 | 9 | 16 | 25 | 3 | 12 | 20 | 9 | 16 | 25 | 6 | 12 | 20 | 6 | 8 | 20 | 6 | 8 | 10 |
| 6 | 1 | 1 | 4 | 1 | 1 | 4 | 1 | 1 | 8 | 1 | 1 | 10 | 1 | 1 | 8 | 1 | 1 | 10 | 1 | 1 |
| 6 | 1 | 1 | 4 | 1 | 1 | 4 | 1 | 1 | 8 | 1 | 1 | 10 | 1 | 1 | 8 | 1 | 1 | 10 | 1 | 1 |
| 25 | 1 | 1 | 20 | 1 | 1 | 20 | 1 | 1 | 25 | 1 | 1 | 25 | 1 | 1 | 20 | 1 | 1 | 15 | 1 | 1 |
| 6 | 12 | 25 | 9 | 16 | 25 | 3 | 12 | 20 | 9 | 16 | 25 | 6 | 12 | 20 | 6 | 8 | 20 | 6 | 8 | 10 |
| 6 | 1 | 1 | 4 | 1 | 1 | 4 | 1 | 1 | 8 | 1 | 1 | 10 | 1 | 1 | 8 | 1 | 1 | 10 | 1 | 1 |
| 6 | 1 | 1 | 4 | 1 | 1 | 4 | 1 | 1 | 8 | 1 | 1 | 10 | 1 | 1 | 8 | 1 | 1 | 10 | 1 | 1 |
| 25 | 1 | 1 | 20 | 1 | 1 | 20 | 1 | 1 | 25 | 1 | 1 | 25 | 1 | 1 | 20 | 1 | 1 | 15 | 1 | 1 |
| 6 | 12 | 25 | 9 | 16 | 25 | 3 | 12 | 20 | 9 | 16 | 25 | 6 | 12 | 20 | 6 | 8 | 20 | 6 | 8 | 10 |
| 6 | 1 | 1 | 4 | 1 | 1 | 4 | 1 | 1 | 8 | 1 | 1 | 10 | 1 | 1 | 8 | 1 | 1 | 10 | 1 | 1 |
| 6 | 1 | 1 | 4 | 1 | 1 | 4 | 1 | 1 | 8 | 1 | 1 | 10 | 1 | 1 | 8 | 1 | 1 | 10 | 1 | 1 |
| 25 | 1 | 1 | 20 | 1 | 1 | 20 | 1 | 1 | 25 | 1 | 1 | 25 | 1 | 1 | 20 | 1 | 1 | 15 | 1 | 1 |
