# Supplementary material for: Mapping potential risks for the transmission of spotted fever rickettsiosis: The case study from the Rio de Janeiro state, Brazil
Source: PLoS One. 2022 Jul 6;17(7):e0270837. doi: 10.1371/journal.pone.0270837 (PMC9258828; doi:10.1371/journal.pone.0270837)
Supplement: S1 File — (PDF) [file pone.0270837.s006.pdf]

BASE TEMÁTICA VETORIAL 1:1.000.000 – RIO DE JANEIRO – COBERTURA E USO DA TERRA - 2014 - GRADE ESTATÍSTICA

O Monitoramento da Cobertura e Uso da Terra do Brasil tem como objetivo espacializar e quantificar a cobertura e uso da terra de todo o território brasileiro a cada dois anos, permitindo a análise da dinâmica do território em termos dos processos de ocupação, da utilização da terra e de suas transformações. O trabalho resulta da interpretação de imagens de satélite, além de informações complementares e levantamentos de campo em todo o país.

Desde o lançamento dos primeiros resultados, uma série de aprimoramentos metodológicos vêm sendo realizados, tais como: adoção da Grade Estatística do IBGE (1km²) como unidade espacial básica de análise e divulgação dos dados; substituição das imagens de satélite MODIS por imagens LANDSAT 8; e alterações nas classes de cobertura e uso da terra.

A Grade Estatística traz como principais vantagens a estabilidade espaço-temporal e a possibilidade de integração e comparabilidade de dados geoespaciais e estatísticos. O uso da grade permite uma independência em relação aos limites político-administrativos, os quais estão frequentemente sujeitos a alterações. As possibilidades de integração vão desde mapas até dados alfa-numéricos de pesquisas demográficas, agropecuárias, entre outras. As imagens de satélite Landsat 8 possuem resolução espacial maior do que as imagens MODIS, ou seja, possuem mais detalhes e possibilitam melhor distinção entre os diversos elementos da superfície terrestre na interpretação visual das imagens, resultando em maior acurácia dos mapas. As alterações nas classes de cobertura e uso da terra visam a simplificação da legenda e compatibilização com diversas classificações nacionais e internacionais. As classes adotadas neste mapa são: Área artificial; Área agrícola; Pastagem com manejo; Mosaico de ocupações em área florestal; Silvicultura; Vegetação florestal; Área úmida; Vegetação campestre; Mosaico de ocupações me área campestre; Corpo d’água continental; Corpo d’água costeiro e Área descoberta.

A série histórica do mapeamento (2000, 2010, 2012 e 2014) sofreu ajustes visando a adequação a nova metodologia e a harmonização com os dados de 2016.

Simples

**Data (Publicação)**	2017-12-28
**Status**	Concluído

Originador

Coordenação de Recursos Naturais e Estudos Ambientais – CREN (IBGE) - Diretoria de Geociências (IBGE/DGC)
Rio de Janeiro , 20031-170 , BR
21 2142 4581
21 2142 0005

**Palavras-chave (Tema)**	• Uso e Cobertura da terra<br>• Grade estatística 1x1km<br>• Rio de Janeiro
**Tipo de representação espacial**	Vetor
**Denominador**	1000000
**Idioma**	por
**Codificação de caracteres**	UTF8
**Categoria temática**	

-20.73 N	-23.39 S	-40.93 E	-44.92 W

Extensão vertical

**Valor mínimo**	0
**Valor máximo**	0

**Identificador de sistema de referência**	GCS_WGS_1984; DATUM: Word Geodetic System 1984
**Forma de distribuição**	
**Protocolo**	WWW:LINK-1.0-http--link
**Recursos online**	http://www.metadados.geo.ibge.gov.br/geonetwork_ibge/srv/en/resources.get?uuid=2c2589d2-e0c4-4900-9a62-870f2994b1fd&fname=&access=private (WWW:DOWNLOAD-1.0-http--download)
**Recursos online**	http://www.geoservicos.ibge.gov.br/geoserver/CREN/wms?service=WMS&version=1.1.0&request=GetMap&layers=CREN:Cobertura_uso_terra_2014_RJ&styles=&bbox=-44.8916563349999,-23.367408859,-40.955901589,-20.7601281999999&width=512&height=339&srs=EPSG:4674&format=application/openlayers (OGC:WMS-1.1.1-http-get-map)

Distribuidor

Fundação Instituto Brasileiro de Geografia e Estatística – IBGE - Centro de Documentação e Disseminação de Informações – CDDI
Rio de Janeiro , 20271-201 , BR
55(21)0800 218181
55(21) 2142-4723

**Nível hierárquico**	Conjunto de dados
**Instrução**	Informações de cobertura e uso da terra obtidas através da reavaliação da discretização na grade estatística da classificação semi-automática de imagens do satélite Terra, sensor Modis, com resolução de 250 a 500 m e do satélite Landsat 8, sensor OLI, com resolução de 30 m aprimoradas com dados auxiliares de campo e de gabinete, compatíveis com a escala 1:1.000.000. A seleção das células foi realizada com base no critério de predominância da área estadual no interior da célula, cada célula pertencendo a um único estado. As classes da legenda aparecem na tabela de atributos de forma codificada, de acordo com a seguinte correspondência: Área Artificial (1), Área Agrícola (2), Pastagem com Manejo (3), Mosaico de Ocupações em Área Florestal (4), Silvicultura (5), Vegetação Florestal (6), Área Úmida (9), Vegetação Campestre (10), Mosaico de Ocupações em Área Campestre (11), Corpo d'Água Continental (12), Corpo d'Água Costeiro (13) e Área Descoberta (14).
**Identificador do arquivo**	2c2589d2-e0c4-4900-9a62-870f2994b1fd XML
**Idioma**	por
**Codificação de caracteres**	UTF8
**Data dos metadados**	2021-03-15T09:30:42
**Nome da norma dos metadados**	ISO 19115:2003/19139
**Versão da norma dos metadados**	1.0

Autor

Instituto Brasileiro de Geografia e Estatística - IBGE - Diretoria de Geociências - DGC / Coordenação de Recursos Naturais e Estudos Ambientais - CREN
Rio de Janeiro , 20031-170 , BR
55(21)2142 4581
55(21)2142 0005

Visões gerais

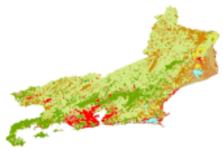

thumbnail

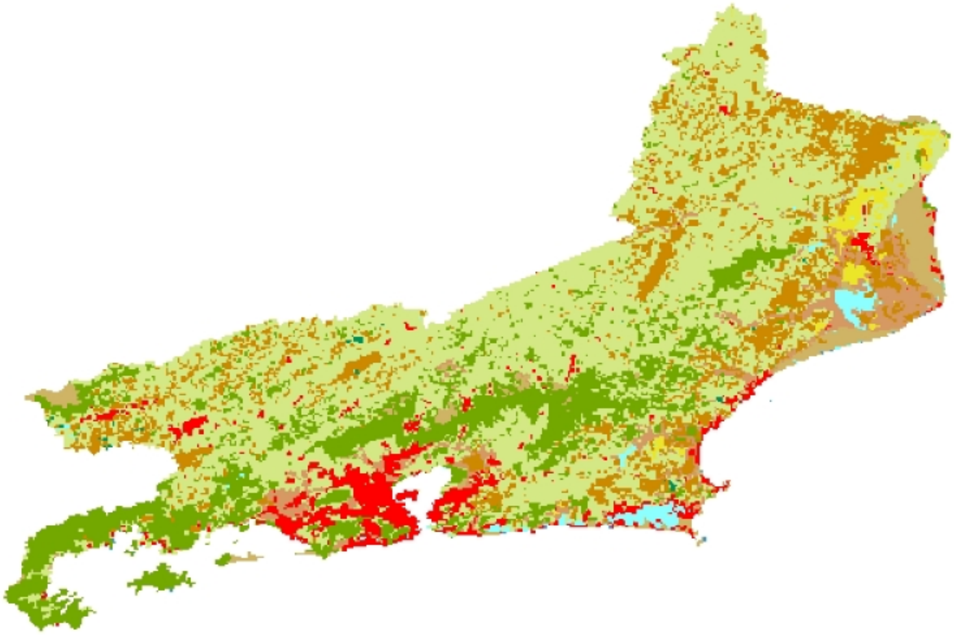

large_thumbnail

Providenciado por

IBGE
