# Supplementary material for: Mapping potential risks for the transmission of spotted fever rickettsiosis: The case study from the Rio de Janeiro state, Brazil
Source: PLoS One. 2022 Jul 6;17(7):e0270837. doi: 10.1371/journal.pone.0270837 (PMC9258828; doi:10.1371/journal.pone.0270837)
Supplement: S2 File — (PDF) [file pone.0270837.s007.pdf]

INDE - Catálogo de Metadados Geográficos

Regiões Geográficas Imediatas do Estado do Rio de Janeiro

Mapa desenvolvido pela Coordenação de Geografia, IBGE. Este mapa retrata a Divisão Regional do Estado do Rio de Janeiro em Regiões Geográficas de Imediatas e Intermediárias 2017.

Simples

**Data (Publicação)**	Junho 2017
**Status**	Concluído

Originador
Centro de Documentação e Disseminação da Informação- CDDI/Coordenação de Projetos Especiais - COPES - Centro de Documentação e Disseminação da Informação- CDDI/ Coordenação de Projetos Especiais - COPES
Rio de Janeiro , 20271-205 , BR
55 (21) 0800 7218181
55 (21) 2142-4723

**Palavras-chave**	• Divisão Regional do Brasil<br>• Regiões Geográficas<br>• Regiões Geográficas Intermediárias<br>• RGInt<br>• Regiões Geográficas Imediatas<br>• RGI<br>• Rio de Janeiro
**Tipo de representação espacial**	Vetor
**Denominador**	2500000
**Idioma**	por
**Codificação de caracteres**	UTF8
**Categoria temática**	

5.27 N	-33.75 S	-28.84 E	-73.99 W

Extensão vertical

**Identificador de sistema de referência**	SIRGAS 2000
**Forma de distribuição**	
**Protocolo**	WWW:LINK-1.0-http--link
**Recursos online**	http://www.metadados.geo.ibge.gov.br/geonetwork_ibge/srv/en/resources.get?uuid=515e67ee-ba6a-417b-bf53-511e5825d7cd&fname=&access=private (WWW:DOWNLOAD-1.0-http--download)
**Recursos online**	http://www.geoservicos.ibge.gov.br/geoserver/CGEO/wms?service=WMS&version=1.1.0&request=GetMap&layers=CGEO:RG2017_regioesgeograficas_rj&styles=&bbox=-44.889320551,-23.368931963,-40.9585185179999,-20.763205462&width=512&height=339&srs=EPSG:4674&format=application/openlayers (OGC:WMS-1.1.1-http-get-map)

Distribuidor
Centro de Documentação e Disseminação da Informação- CDDI - Fundação IBGE -Instituto Brasileiro de Geografia e Estatística
Rio de Janeiro , 20271-205 , BR
55 (21) 0800 7218181
55 (21) 2142-4723

**Nível hierárquico**	Conjunto de dados
**Instrução**	"Fonte: IBGE- Malha Municipal, 2015; Base Cartográfica Contínua do Brasil, ao<br><br>Milionésimo - BCIM 2010; SRTM- Relevo sombreado, 2000."
**Identificador do arquivo**	515e67ee-ba6a-417b-bf53-511e5825d7cd XML
**Idioma**	por
**Codificação de caracteres**	UTF8
**Data dos metadados**	2017-05-23T16:45:47
**Nome da norma dos metadados**	ISO 19115:2003/19139
**Versão da norma dos metadados**	1.0

Fornecedor de recurso
Instituto Brasileiro de Geografia e Estatística - Coordenação de Geografia/DGC/IBGE
Rio de Janeiro , 20031170 , BR
55 (21) 0800 7218181
55 (21) 2142-4723

Visões gerais

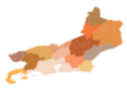

thumbnail

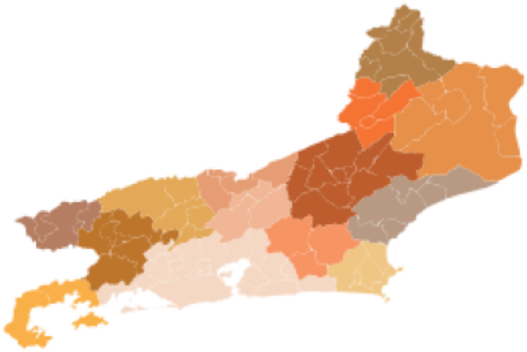

large_thumbnail

Providenciado por
