# Supplementary material for: Mapping potential risks for the transmission of spotted fever rickettsiosis: The case study from the Rio de Janeiro state, Brazil
Source: PLoS One. 2022 Jul 6;17(7):e0270837. doi: 10.1371/journal.pone.0270837 (PMC9258828; doi:10.1371/journal.pone.0270837)
Supplement: S3 File — (PDF) [file pone.0270837.s008.pdf]

Atlas do Censo Demográfico 2010 - Unidades da Federação - 2010

Divisão Política.

Recorte territorial por Unidades da federação e grandes regiões.

Fonte: IBGE, Censo Demográfico 2010.

Simples

**Data (Publicação)**	28/06/2013
**Status**	Concluído

Originador

Diretoria de Geociências - DGC / Coordenação de Geografia - CGEO - Fundação Instituto Brasileiro de Geografia e Estatística - IBGE
Rio de Janeiro , 20031-170 , BR
55 (21) 2142-4578
55 (21) 2142-0574

**Palavras-chave (Tema)**	• Atlas do Censo Demográfico 2010
**Palavras-chave (Tema)**	• Divisão político-administrativa
**Palavras-chave (Tema)**	• Unidade da Federação
**Tipo de representação espacial**	Vetor
**Denominador**	2500000
**Idioma**	por
**Codificação de caracteres**	UTF8
**Categoria temática**	

5.27	N	-33.74	S	-34.79	E	-74.00	W

Extensão vertical

**Valor mínimo**	0
**Valor máximo**	0

**Identificador de sistema de referência**	SIRGAS2000
**Forma de distribuição**	
**Recursos online**	IBGE - Atlas do Censo Demográfico 2010 (WWW:LINK-1.0-http--link)
**Recursos online**	http://www.metadados.geo.ibge.gov.br/geonetwork_ibge/srv/en/resources.get?uuid=3bba887e-6cb1-4a7d-83bd-9906b832d81a&fname=&access=private (WWW:DOWNLOAD-1.0-http--download)
**Recursos online**	http://www.geoservicos.ibge.gov.br/geoserver/CGEO/wms?service=WMS&version=1.1.0&request=GetMap&layers=CGEO:C02_unidades_da_federacao_2010&styles=&bbox=-73.9904499689983,-33.7520812705959,-28.8360947061847,5.27184107724553&width=512&height=442&srs=EPSG:4674&format=application/openlayers (OGC:WMS-1.1.1-http-get-map)

Distribuidor
Centro de Documentação e Disseminação de Informações - CDDI - Fundação Instituto Brasileiro de Geografia e Estatística - IBGE
Rio de Janeiro , 20271-201 , BR
55 (21) 0800 218181
55 (21) 2142-4723

**Nível hierárquico**	Serviço
**Instrução**	Dados do Censo Demográfico 2010; Malha Municipal produzida pela DGC/CETE-IBGE; Base cartográfica produzida pela DGC/CCAR-IBGE.
**Identificador do arquivo**	3bba887e-6cb1-4a7d-83bd-9906b832d81a XML
**Idioma**	por
**Codificação de caracteres**	UTF8
**Data dos metadados**	2015-08-28T16:50:36
**Nome da norma dos metadados**	ISO 19115:2003/19139
**Versão da norma dos metadados**	1.0

Autor
Diretoria de Geociências - DGC / Coordenação de Geografia - CGEO - Fundação Instituto Brasileiro de Geografia e Estatística - IBGE
Rio de Janeiro , 21241-051 , BR
55 (21) 2142-4987
55 (21) 2142-4973

Visões gerais

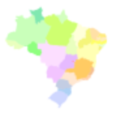

thumbnail

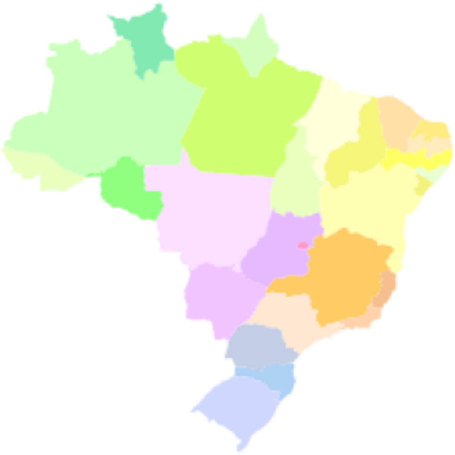

large_thumbnail

Providenciado por

IBGE
