# Supplementary material for: Mapping potential risks for the transmission of spotted fever rickettsiosis: The case study from the Rio de Janeiro state, Brazil
Source: PLoS One. 2022 Jul 6;17(7):e0270837. doi: 10.1371/journal.pone.0270837 (PMC9258828; doi:10.1371/journal.pone.0270837)
Supplement: S4 File — (PDF) [file pone.0270837.s009.pdf]

Consultar Manifestação

Respostas

26/04/2018 11:45

Tipo

Resposta Conclusiva

Responsável

Departamento de Vigilância das Doenças Transmissíveis

Decisão

Acesso Concedido

Especificação da decisão

Resposta solicitada inserida no Fala.Br

Destinatário Recurso 1ª

Prazo para recorrer

07/05/2018

Anexos 1-Excel-Base-dados-Sinan-Net-Febre-maculosa-2007-2016-RJ-MG-SP.xlsx 2-Febre_Maculosa_v5.pdf 3-DIC_DADOS_Febre_Maculosa_v5.pdf 4-Febre_Maculosa_v5_instr.pdf

Prezado cidadão, informamos abaixo a resposta à sua solicitação, no prazo da lei de acesso a informação.

Em anexo envio a: 1) base de dados (em Excel) com as notificações de febre maculosa, não nominal, de 2007 a 2016 para o estado do rio de janeiro, minas gerais e São Paulo.
Adicionalmente envio a: 2) ficha de notificação e investigação, 3) dicionário de dados e 4) instrutivo de preenchimento.
Estes documentos auxiliares (2,3 e 4) poderão apoiar o cidadão nas referidas análises que pretende realizar.

Estamos à disposição para quaisquer esclarecimentos.

Teor

Resumo
DADOS EPIDEMIOLÓGICOS (SINAN) FEBRE MACULOSA NOTIFICADOS ENTRE 2007 E 2016 PARA DETERMINADOS MUNICÍPIOS DO RJ, MG E SP
Fale aqui
Solicito acesso, sem a identificação dos pacientes,dos dados epidemiológicos a respeito da Febre Maculosa dos casos notificados e confirmados (SINAN) entre 2007 e 2016 nos municípios relacionados nas planilhas em anexo (municípios de residência e municípios de notificação). Os dados serão utilizados em análise epidemiológica com fins acadêmicos no meu projeto de doutorado que tem como orientadores Lena Geise - UERJ e Gilberto Salles Gazeta - Fiocruz, e como um dos objetivos de pesquisa a ecoepidemiologia da FM na bacia do rio Paraíba do Sul. ,

Anexos Originais

Nome
MUN RESIDENCIA FM 2007 A 2016.xlsx
MUN NOTIFICACAO FM 2007 A 2016.xlsx

Manifestação

Tipo de manifestação
Acesso à Informação
Número

25820.001767/2018-61
Esfera
Federal
Órgão destinatário
MS – Ministério da Saúde

Serviço
-
Órgão de interesse
-
Assunto
Outros em Saúde
Subassunto
Tag
-

Data de cadastro
24/03/2018
Prazo de atendimento
26/04/2018
Situação
Concluída
Registrado por
CLAUDIO MANUEL RODRIGUES
Modo de resposta
Pelo sistema (com avisos por email)
Canal de entrada
Internet

Anexos

Anexos da Manifestação

Origem	Nome	Extensão
Anexo Manifestação	MUN RESIDENCIA FM 2007 A 2016.xlsx	xlsx
Anexo Manifestação	MUN NOTIFICACAO FM 2007 A 2016.xlsx	xlsx
Anexo Resposta	1-Excel-Base-dados-Sinan-Net-Febre-maculosa-2007-2016-RJ-MG-SP.xlsx	xlsx
Anexo Resposta	2-Febre_Maculosa_v5.pdf	pdf
Anexo Resposta	3-DIC_DADOS_Febre_Maculosa_v5.pdf	pdf
Anexo Resposta	4-Febre_Maculosa_v5_instr.pdf	pdf

Download

Históricos de ações
