# Supplementary material for: Mapping potential risks for the transmission of spotted fever rickettsiosis: The case study from the Rio de Janeiro state, Brazil
Source: PLoS One. 2022 Jul 6;17(7):e0270837. doi: 10.1371/journal.pone.0270837 (PMC9258828; doi:10.1371/journal.pone.0270837)
Supplement: S5 File — (PDF) [file pone.0270837.s010.pdf]

Dear Marcelo Eduardo Dantas (CPRM)

I request permission for the open-access journal PLOS ONE to publish **Geoenvironmental Map of the Rio de Janeiro state** under the Creative Commons Attribution License (CCAL) CC BY 4.0 (http://creativecommons.org/licenses/by/4.0/). Please be aware that this license allows unrestricted use and distribution, even commercially, by third parties. Please reply and provide explicit written permission to publish **Geoenvironmental Map of the Rio de Janeiro state** under a CC BY license and complete the attached form.

Best regards,

Claudio Manuel Rodrigues

Request for Permission to Publish Content under CC-BY License

Dear Rights Holder or Representative,

I have submitted a paper for publication in a PLOS journal, and wish to include the content listed below in the paper. I'm hereby requesting your (or your company's or institution's) permission to include the content in my paper. Please note that all PLOS journals are published under a Creative Commons Attribution License (CC BY), which allows for unrestricted use and distribution, even commercial, as long as attribution is given to the creator or rights holder of the content. See https://creativecommons.org/licenses/by/4.0/.

To grant me permission to use the content in my PLOS paper, please fill in the information below and then scan the completed form and send it to me at my email address.

Thank you.

My name:

Cláudio Manuel Rodrigues

My email address:

Rua Haddock Lobo 61, cob 01, Estácio, Rio de Janeiro, RJ, Brazil, CEP 20260-130

Description of the content which I'm seeking permission to use (citation and/or title, and pasted screen shot, if applicable):

Shapefile vector files related to the "Geoenvironmental Map of the Rio de Janeiro state"

Link to the Content:

http://tiny.cc/9blnuz

* * *

On behalf of myself or the rights holder, I hereby grant the permission sought herein.

Signature of Party Granting Permission:

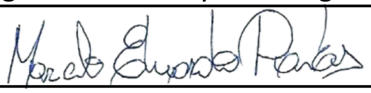

Date:

17/01/2022

Printed Name and Title:

Marcelo Eduardo Dantas, MSc. in Geography, Rio de Janeiro Federal University.
Executive Coordinator - DEGET. Geological Survey of Brazil – SGB/CPRM.
