# Supplementary material for: Mapping potential risks for the transmission of spotted fever rickettsiosis: The case study from the Rio de Janeiro state, Brazil
Source: PLoS One. 2022 Jul 6;17(7):e0270837. doi: 10.1371/journal.pone.0270837 (PMC9258828; doi:10.1371/journal.pone.0270837)
Supplement: Attachment — Submitted filename: Response to Reviewers.pdf [file pone.0270837.s011.pdf]

Reply Letter

PONE-D-21-18008

Date: 02-01-2022

Dear editor and reviewers,

Upon receiving the reviewers' suggestions, we observed how opportune the reformulation was, not only of a group of sentences, but of considerable parts of the manuscript in question, since we understand it as a (good) challenge to bring up the issues related to the organization of our scientific thinking not yet laid out in the text. We bet on the reorganization of the Materials and methods section in partitions that would facilitate the understanding of the step-by-step that we built to reach the results. We also chose to add more information to the Introduction and reformulate the Results and Discussion sections in order to offer readers different perspectives related to vector-host interactions demonstrated in each of the potential hazard maps produced. For a better understanding of how much we worked on the new writing of the manuscript, we offer below a list of responses to the reviewers' suggestions.

#Reviewer 1

In general:

1. The main weakness of the article is that the author did not do a very good job of explaining the methods
   Answer: We rewrite the section in question to give more clarity to the method used in the study. We have separated the section into partitions that best express the methodological design of the study.

2. Loss of opportunity to deepen and use land use data to understand the risk of rickettsiosis being associated with human-dominated landscapes.
   Answer: By rewriting the study we tried to give the reader a better understanding of the relationships between vectors and hosts with the geographic layers chosen to produce the maps of potential risk.

3. To improve methods, add subheadings.
   Answer: Suggestion accepted. We made uses of subheadings in the section.

4. Grouping cases based on milder (*Rickettsia parkeri*) and more severe (*Rickettsia rickettsii*) symptoms can lead to inaccurate conclusions and affect results. The data is still interesting, but it is necessary to at least point this out as one of the main limitations of the study and how testing humans by serological

tests, rather than using molecular approaches, can lead to a cross-reaction between Rickettsia species.

Answer: In this revised version of the manuscript, we seek to offer a broader view of the distinction between rickettsial species involved in spotted fever transmission in Rio de Janeiro state. We understand that the use of epidemiological data is essential to distinguish between possible etiological agents, since the official laboratories of the Brazilian national epidemiological surveillance network do not distinguish between the species involved, only focusing on the diagnosis of the genus of the bacterium that causes the disease. It was observed that the gold standard for diagnosing rickettsiosis is the indirect immunofluorescence test, which, despite having high specificity, can actually lead to cross-reactions between rickettsiae of the same group (heterotypic antibodies), as is the case with *Rickettsia rickettsii* and *Rickettsia parkeri*.

TITLE

1. Remove "proposal"
   Answer: Suggestion accepted.

2. Spotted fever group? Or spotted fever rickettsiosis? I suggest clarifying what they are referring to. Suggestion: use the name of the pathogen instead of the diseases, as it is the pathogen that is actually transmitted.

Answer: Yes, the idea is to work with spotted fever rickettsiosis. Alerted to this possible conceptual confusion, we chose, throughout the text, to clearly express our intention. We work with the pathogens, but also with the diseases linked to them (*Rickettsia rickettsii* -> BSF rickettsiosis and *Rickettsia parkeri* -> SF rickettsiosis).

ABSTRACT

1. General comments: Key results and how they contribute to the system are missing. The authors mentioned that there were risk and protective factors for spotted fever rickettsiosis, but failed to mention what they were.

   Answer: We follow the suggestion

2. Line 86: Spotted fever rickettsiosis is actually a group of diseases and not just an infectious disease. However, whether the authors are referring to Brazilian spotted fever transmitted by R. rickettsii or to all rickettsiosis of the spotted fever group needs to be clarified.

   Answer: We follow the suggestion.

3. Line 92: "risk" rather than "danger"

Answer: Although we consider different concepts terminologically, we follow the suggestion.

INTRODUCTION

In general: the authors have listed several studies on hazard mapping, but it would be great if they could briefly explain what their main findings are and how they contribute to knowledge and better understanding of risk factors for pathogen transmission.

Answer: we added a paragraph to the text referring specifically to the three previously cited articles.

1. Line 119-121: Suggestion: clarify which ticks are the main vectors of R. parkeri and R. rickettsii.

Answer: Suggestion accepted.

2. Line 121: "genus" instead of "genre"

Answer: Adjusted.

3. Line 126-127: The instruction is confusing. Please clarify.

Answer: The text was rewritten in order to give more clarity to the message.

MATERIAL AND METHODS

1. Line 145-150: It's not clear where the data comes from and how the authors got the data from vectors and hosts. In addition, data on ticks and hosts associated with Rickettsia spp. presence? It is important that authors explain how human rickettsiosis cases were detected (e.g. molecular, serological).
   Answer: We sought to rewrite the text to clarify the origin of the data used in the analysis related to vectors, hosts and rickettsiosis occurrences in humans. It is not possible to obtain data on rickettsial infection in ticks related to all suspected cases in humans, as these vectors are not collected by local environmental health surveillance services other than after laboratory diagnosis or through deduction by the medical clinic and association with the probable site of infection by the local epidemiological surveillance service. That is why we make use of the approximation of the more or less severe clinical presentation with the possible etiologic agent involved. At one point in the text (Epidemiological Validation) we have the information that the gold test for the disease in Brazil is the indirect immunofluorescence reaction of paired samples (serological), but it is also possible to perform molecular analysis from the Polymerase Chain Reaction (PCR).

2. Line 184-186: It seems that the authors use the presence of certain tick species and hosts, but do not explain why they chose these species and on the basis of what values were assigned to each.
   Answer: The entire section was rewritten, reorganized in its presentation, to clarify the methodological design adopted and to provide robustness to the results found. In addition, as a supplementary material, the set of spreadsheets that offers the calculation and operationalization mechanisms of the adopted weighting models was offered.

3. Line 186: What values 1 and 5 represent? Please clarify
   Answer: As already mentioned, the section was reorganized to provide clarity on the methodological design adopted and to provide robustness to the results found.

4. Line 211-213 (Table 2): The table was written in Portuguese and not in English.
   Answer: Adjusted.

5. Line 225: Please explain what was the nosological classification of the disease?
   Answer: The word "nosological" was used arbitrarily in order to "characterize" or "classify" the disease, but it does not seem to be the best way to express our thoughts. Therefore, it was replaced to give more clarity to the text, using the expression "symptom severity", which, together with the new wording of the section, seemed more appropriate.

RESULTS

1. It seems that the authors failed to explain all the results, like figures 2 and 3, it also seems that figure 4 should be before figures 2 and 3, as they describe the study area.
   Answer: As already said, by reorganizing the section, we believe that we have solved comprehension problems such as this statement. Some figures have been replaced, others redone or included in the course of the manuscript to give a better understanding of what we propose and to give fluidity to the reading.

DISCUSSION

1. Line 358: use “rickettsiosis”

   Answer: Adjusted.

Line 376-377: host and Rickettsiosis

   Answer: Adjusted.

2. Line 376-380: It's hard to follow.

Answer: The text was rewritten in order to give more clarity to the message.

Reviewer #2:

1. The results found in the article need to be described in more detail, as well as the discussion, in which the data discussed are not fully consistent, especially in the choice of vertebrates and possible amplified animals that were used as a variable to classify the risk area.
   Answer: We accepted the criticism in a positive way, proposing a complete restructuring of the Results section, describing them in greater detail, taking care not to be too boring to give fluidity to the reading. The Discussion section was added with paragraphs that, in addition to seeking to give more emphasis to the results found, tried to achieve the desired consistency, including the choice of the four vectors and the three representatives of hosts used as a variable to classify the area of potential risk for rickettsiosis.

2. It is also interesting to detail what led to the classification of the disease in two forms (BSF and SF) and the possible vectors that were also chosen as a variable to classify the area as at risk.
   Answer: We rewrote the manuscript in order to offer an adequate explanation of the eco-epidemiological criteria that guide our methodological approach and that led us to classify the disease in two forms (BSF and SF), in addition to expressing more clearly the perspective of choosing the four tick species and three vertebrate hosts used as a variable to classify the area as a potential risk for rickettsiosis. We hope we got it right.

3. *Amblyomma aureolatum* is missing from the discussion of the results.
   Answer: Adjusted.

Best regards,

The authors
