# Supplementary material for: Mapping potential risks for the transmission of spotted fever rickettsiosis: The case study from the Rio de Janeiro state, Brazil
Source: PLoS One. 2022 Jul 6;17(7):e0270837. doi: 10.1371/journal.pone.0270837 (PMC9258828; doi:10.1371/journal.pone.0270837)
Supplement: Attachment — Submitted filename: Response Letter to Reviewers.docx [file pone.0270837.s012.docx]

**Response Letter to Reviewers**

Reviewer #1: The authors improved the manuscript. However, there are still some issues that need to be fixed.

a. We thank you for your kind observation. We were challenged to improve our academic writing to seek to clarify to the reader the motivations of our study, whether qualifying the introduction, reorganizing the methodology into legends or developing images to give a better understanding of our results.

My main concern that I did not think about on my first review, is that I assumed the experts knew where the cases were reported, so when they gave the score values to the cover and land use and vector-host association it was likely that the spatial risk model would match where the cases are, potentially biasing the model results. I think it would be more valuable if the authors can discuss more about whether the risk maps are associated with the distribution or density of vectors and/or hosts, rather than the human cases.

b. The panel of experts was designed from the perspective of:

1. All participants are experts in different areas of knowledge, which, when associated, could offer a more reliable result of the relationship between vectors and hosts with components related to soil cover and the geoenvironmental aspects of the study territory;
2. we expected the experts to analyze the ecological relationship between the animals that participate in the ecological cycle associated with rickettsiosis, that is, suspected or confirmed cases of the disease would not be observed in the procedures related to the expert panels; and
3. we did not control for this hypothetical bias, in which experts would be influenced by recognizing the points (exact or not) of infection (human cases), since, in fact, we will hardly have in the disease investigation sheets the exact location of contact with infected ticks (this yes, a problem to be solved by the management of health surveillance) and, certainly, very few (or none?) of these specialists would have access to investigation files because they are reserved for epidemiological and environmental surveillance of Brazilian institutional services.

c. The model we proposed is related to the spatial availability of the host / vector distribution. This is clear in the first paragraph to Methodological proposal:

"Understanding that the potential risk for the transmission of spotted fever rickettsiosis (PRSF) can be demonstrated by the relationship between vectors (v) and hosts (h) projected in the geographical space, we formulated a methodological proposal".

**ABSTRACT**

Line 94: Please describe which ones were the risk and protection factors. Based on what the authors studied it seems the statement is not accurate, since the authors modeled only the risk spatially based on scores previously given to the association variables, but they do not aim to study the influence of those factors in the cycle.

d. We offer an editing of the manuscript text for better understanding of the reader:

“The results achieved were substantially encouraging, considering that there are territories with greater or lesser expectation of risk for spotted fever in the study area”.

Line 99: Sceneries is incorrect

e. Corrected in the text (“scenery”)

**INTRODUCTION**

Line 111: Spotted fever rickettsiosis is caused by a group of species

f. We appreciate the suggestion, but we believe that the text is consistent with what we communicate to the reader. In the immediate sequence of the text, we identified that there is a group of vector species that transmit bacteria of the genus *Rickettsia*, etiological agents of Spotted Fever (SF) and Brazilian Spotted Fever (BSF).

Line 115: humans instead of human beings.

g. Corrected in the text (“human ~~beings~~”)

Line 122-127: seems that the explanation of choosing ticks would fit better in the methods section.

h. Corrected in the text (“~~Considering this we selected~~ Three tick species of the…”)

**METHODS:**

The methods are still a little bit hard to follow. For example, the authors did not mention how many types of landscape/habitats are within the study in the manuscript. They only mentioned them in the table 2. Same happens with geoenvironmental domains. I think the authors should briefly explain what they are in the methodological proposal section.

i. We emphasize that the characteristics related to land use and geoenvironmental domains are displayed in Figure 2 which, coupled with the legend and associated with the image in Figure 1, offers a view of both profiles in the study territory. In addition, we leave the access links to more information about it and the reference literature available in the manuscript text.

We understand that the reviewer's observation must be taken into account and, therefore, we offer an editing of the manuscript text for better understanding of the reader:

“By definition, geoenvironmental domains represent morphostructures that relate to remarkable events, which are responsible for the current arrangement of the relief and for the less mutable characteristics of the landscape. From the perspective of our study, the geoenvironmental domains refer to a larger taxon, compatible with regions with the same geological and geomorphological characteristics that group the same habitats and their respective faunal and floristic communities [19].

The geoenvironmental data used **in this work** are the result (…)”

Line 276: I would suggest adding subtitles to the Expert panels section. It is hard to follow all weighting procedures.

j. At the end of the first paragraph of the Expert Panel subtitle, we made a small edit in order to give more elegance to the text, but we understanding that two expert panels were held at different times, both portrayed in the Methodology Section. The first panel [line 306: "This primary weighting (...)"], which has the result portrayed in Table 1, used 16 panelists from different areas of knowledge and with experience related to rickettsial diseases that we discussed during the study. The methodological perspective is well described, paragraph after paragraph. The second panel [line 347: "In a second moment, four specialists(...)"] had 4 experts and developing the methodological procedures laid out in the text, reached the results expressed in Tables 2 and 3. Both panels are documented in supplemental material provided by the authors. Therefore, we consider that the text is clear and the use of subheadings would not add better understanding for the reader.

Line 288: I wonder if there was any way to confirm the values giving by the experts base on reviewing the literature.

k. The choice to consult the specialists stems from the fact that there is a scarce literature focused on the ecological theme of the enzootic and epizootic cycles of rickettsiosis in the study area. Of the few epidemiological studies published in the last five years and related to the study area, we highlight the themes of altitudinal distribution of tick species of importance for the transmission of SF and BSF (https://doi.org/10.1186/s13071-022-05250 -6); factors related to animal hosts of certain tick species on Ilha Grande (https://doi.org/10.1080/01647954.2020.1804999); and eco-epidemiological scenario of spotted fever (https://doi.org/10.3389/fmicb.2017.00505). Thus, the methodological profile was structured in such a way as to make scientific arguments for use in the study based on the opinion of experts, who accumulate knowledge during their academic careers.

This question is also related to the fact that capybara scores showed to be more likely in urban areas than rural areas. Does this make sense? If so, could you please explain it why?

l. Like horses and domestic dogs, capybaras deserved to receive higher scores among the specialists consulted because, in the case of Rio de Janeiro state, they are present in urbanized areas of territory. It is noted that Rio de Janeiro state has a historical process of urbanization, since the time of Colonial-Brazil, it had economic, cultural and political importance, becoming the capital of the country for many centuries until, in the 1960s, the inauguration of Brasília, DF took place.

Dogs play an important role in the social life of humans, having been with us since they approached and were domesticated by the peoples of Southeast Asia over 30,000 years ago ([www.sciencedaily.com/releases/2017/07/170718113516.htm](http://www.sciencedaily.com/releases/2017/07/170718113516.htm)). It is easy to understand that it is present in practically the entire territory studied, thus justifying the weight offered as an important host of ticks of epidemiological importance for rickettsial diseases in the study area.

Horses have a very similar historical perspective to dogs, justifying their presence in rural and peri-urban areas. Culturally, in areas of greater human density, it can be seen as a working animal, in the case of the mounted police, but also related to the low-income population, who live in precarious conditions and use their services for personal transport or household items, as well as scraps of constructions and other objects that can be transported on small metal and wooden boards, being an important source of income for this part of the population (<http://dx.doi.org/10.4102/jsava.v91i0.2009>).

Capybaras accompany the recent urbanization process not only of the study area, but of the entire country (<https://www.bravietour.com.br/mangrove-and-jungle-tour> ; <https://doi.org/10.1016/j.landurbplan.2022.104398>

and <https://link.springer.com/article/10.1007/s10493-012-9533-1>). Specifically in Rio de Janeiro state, urban agglomerations take the place of old dairy farms or small peri-urban swiddens, which were once responsible for supplying vegetables and fruits to cities. Commonly associated with water collections (rivers, lagoons, etc.) and field areas, it is currently common for them to be found walking on the streets of cities in Rio de Janeiro state, including its capital, interacting with the urban landscape and, in a way, quickly adapting to new living conditions by finding food and security necessary to reproduce and generate offspring that guarantee the maintenance of future generations. It can be said currently, in addition to the ecological and environmental perspective, sanitary nuances have been a questioning factor for their presence in inhabited areas, since they are protected from slaughter by national legislation and because, in cities, they do not have natural predators (<http://doi.org/10.1089/vbz.2019.2479> and <https://doi.org/10.1093/jmammal/gyaa144>).

Line 332: T is missing

m. Corrected in the text (“**T**he”)

Line 346: Please clarify what the peculiar characteristics are.

n. We offer an editing of the manuscript text for better understanding of the reader:

“(…) characteristics that differentiate them from each other **(*e.g.*, eating habits; degree of insolation, humidity and temperature; vegetation type; altitudinal range and anthropophilia)**, which (…)”

and respective references [**1**, 10, **11**].

Line 454: Please add a reference for sensitivity and specificity. High specificity means it can differentiate between R. parkeri and R. rickettsii?

o. Thanks for the inquiry. We edited the text for better understanding by the reader. We missed the opportunity to offer a more up-to-date reading of the procedures determined by the reference laboratories in the original text and took advantage of the description of the gold standard diagnosis for spotted fever by the brand-new edition of the Health Surveillance Guide (5th ed.) of 2021, which has just been published by the Ministry of Health, to elucidate the issue. We also updated the guide cited [2] in the References section.

The text was like this:

“(…) This method ~~comprises a high sensitivity and specificity reaction that can be used to identify and quantify specific immunoglobulins of the IgM and IgG class~~ is established by the identification and quantification of specific immunoglobulins of the IgM and IgG class, which increase in titer with the evolution of the disease. **The result should always be interpreted based on the clinical and epidemiological context related to the suspected case, since IgM antibodies can cross-react with other diseases (*e.g.,* dengue and leptospirosis) and, in general, perform better between the seventh and tenth day of illness.** In addition (…)”

Line 474: Is there any reference that support the fact that R. rickettsii is not associated with any mild cases? If so, please add it.

p. To our knowledge, there is no publication that categorically states that there is no possibility of non-severe cases of spotted fever caused by infection with the agent Rickettsia rickettsii. But we also don't know of any scientific publication that denies this possibility in an unquestionable way.

**DISCUSSION**

Line 649: Type

q. Editing: “(...) which seems to be a consequence of the small spread of other etiological agents than *Rickettsia rickettsii*.”

Line 648: It is well known Rickettsia parkeri tend to cause mild cases and R. rickettsii more severe cases. However, I think it would be valuable to discuss about whether R. rickettsii can also be associated with mild cases as well. Assuming that all mild cases are caused by Rickettsia species other than R. rickettsii it may inaccurate.

r. Yes, thanks for your question. Although it is very important for Health Surveillance to clinically recognize the disease, in the study we did not seek to discuss the clinical nuances linked to the different etiological agents that would cause rickettsiosis in the territory of Rio de Janeiro. We focus on producing knowledge about the life cycles of four tick species, and consequently, the rickettsiae related to them in a delimited geographic space, seeking to determine a possible spatial distribution of the potential danger in having contact with the disease.

Currently, in Brazil, it is still a challenge to identify the species related to symptomatic cases of rickettsiosis, since, for purposes of epidemiological surveillance, it is enough to recognize the genus *Rickettsia* and confirm the suspicion notified by the local health management. However, the scientific literature shows that there is a group of rickettsiae that differs from the symptomatology and the medical clinic expected for cases related to *Rickettsia rickettsii*. These are oligosymptomatic cases, at best, cases of mild symptoms that progress very positively to cure, without major complications. In a way, the cases related to *Rickettsia rickettsii* and *Rickettsia parkeri* - Atlantic Forest strain are relatively well delimited spatially and also maintain a distinct correlation with the clinical symptoms presented by the patients.

In fact, there is no way to precisely determine whether all cases related to *Rickettsia rickettsii* are severe enough to lead patients to hospitalization and, sometimes, to death. However, it is recognized that with such distinct clinical characteristics, with such disparate symptoms, we would hardly have cases of FS (associated with *Rickettsia parkeri*) being confused with BSF (associated with *Rickettsia rickettsii*), even more recognized the eco-epidemiological scenery that we described in the study. The confusion could be due to the congruence of areas of occurrence of *Amblyomma ovale*, *Amblyomma aureolatum* and *Amblyomma sculptum* in Rio de Janeiro state and, thus, with the perspective of the circulation of different etiological agents in the territory. Although it is in a relatively small territory, but of high complexity regarding the abundance of natural landscapes and the types of relief demonstrated in the study by the geo-environmental profile and the cover and use of the soil, the study showed that it is possible to identify the areas affected by the types SF and BSF for the methodological proposal that we are offering.

To finish this answer, we explained the limitations of the study, including the aspect mentioned by the reviewer, in the text of the manuscript that we present below:

“As for the validation of the methodology using official data on the occurrence of cases, hospitalizations and deaths from spotted fever rickettsiosis in the study area, we observed that digressions were necessary regarding the classification of the etiological agents in question. It was observed that the laboratory diagnosis offered by the National Network of Epidemiological Surveillance Laboratories, despite having a high power of precision, maintains a standard conduct of classifying the genus (*Rickettsia* spp.) and the identification of the species involved in the occurrence of cases is not routine. It is also important to note that the sites of probable infection are still considered imprecise, despite all efforts by professionals from the local Environmental Surveillance Service to define them better”.

And, even more, in the last paragraph of the Discussion:

”Thus, we chose to make approximations in our analyses, supported by the clinical-epidemiological and environmental diagnoses performed by the local Health Surveillance teams, associating cases considered more serious, in which hospitalization and / or death of the patient occurred, as a proxy for Rickettsia rickettsii infection and, conversely, with no worsening of the clinical condition that would require hospitalization of the patient or that would allow a cure outcome, as a proxy for infection by another rickettsial species, as in the case of *Rickettsia parkeri*. We understand that the option for this methodological design is not a limitation that invalidates or weakens our study, on the contrary, it offers very creative epidemiological contours that indicate the need for more accurate biotic data with the innovation of local environmental and epidemiological surveillance strategies”.

Reviewer #2: After a new evaluation, I recommend the publication because the corrections, suggestions and changes were met by the authors.

s. Thank you for supporting us in this academic effort to disseminate our manuscript.
